# 

**Published:** 1863-04

**Authors:** 


### CHICAGO MEDICAL JOURNAL.

**Terms, $2.00 per Annum.**

### CONTENTS OF THE APRIL NUMBER.

PAGE.

ORIGINAL COMMUNICATIONS.

Directions for Using Bromine. By Dr. M. Goldsmith........................ 145
My Dissection Wound and what Came of it. By R**........................ 147

SELECTED.

Clinical Lecture on the Treatment of Acute Rheumatism, delivered at St. Mary's Hospital, London. By Thos. K. Chambers, M. D.......... 154
Insanity and Intemperance. By Andrew McFarland, M. D................. 162
EDITORIAL AND MISCELLANEOUS.............................................. 183

